# Regulatory Effect of Inflammatory Mediators in Intervertebral Disc Degeneration

**DOI:** 10.1155/2023/6210885

**Published:** 2023-04-17

**Authors:** Zhangfu Li, Honghao Yang, Yong Hai, Yunzhong Cheng

**Affiliations:** Department of Orthopedic Surgery, Beijing Chao-Yang Hospital, Capital Medical University, Beijing 100020, China

## Abstract

Intervertebral disc degeneration (IDD) is a major contributor to back, neck, and radicular pain. It is related to changes in tissue structure and function, including the breakdown of the extracellular matrix (ECM), aging, apoptosis of the nucleus pulposus, and biomechanical tissue impairment. Recently, an increasing number of studies have demonstrated that inflammatory mediators play a crucial role in IDD, and they are being explored as potential treatment targets for IDD and associated disorders. For example, interleukins (IL), tumour necrosis factor-*α* (TNF-*α*), chemokines, and inflammasomes have all been linked to the pathophysiology of IDD. These inflammatory mediators are found in high concentrations in intervertebral disc (IVD) tissues and cells and are associated with the severity of LBP and IDD. It is feasible to reduce the production of these proinflammatory mediators and develop a novel therapy for IDD, which will be a hotspot of future research. In this review, the effects of inflammatory mediators in IDD were described.

## 1. Introduction

Intervertebral disc degeneration (IDD) is a disease of the discs that link adjacent vertebrae, with structural damage leading to a degeneration of the discs and surrounding areas. The intervertebral disc (IVD) is a fibrocartilage tissue that joins the adjacent vertebral bodies in the spine. The nucleus pulposus (NP) is the central component of the IVD and is rich in elastic colloidal compounds, including proteoglycans and type II collagen [1]. IDD can be diagnosed and graded by conventional T2-weighted magnetic resonance images, in which the colour and homogeneity of the disc, distinction of nucleus and annulus, disc signal intensity, and disc height are the basis for grading [2]. IDD is associated with disc herniation, spondylosis, lumbar spinal stenosis, sagittal imbalance of the spinal-pelvic complex, and neurological symptoms, such as low back pain (LBP), limb numbness, and decreased muscle strength [3–5]. The most common symptom of IDD is LBP, which impacts the quality of life of middle-aged and elderly individuals while increasing the economic burden on families and society [6, 7]. Although current evidence-based medicine has identified IDD as the result of a variety of genetic, traumatic, inflammatory, lifestyle, aging, and nutritional variables, the pathogenic processes implicated in the development of IDD remain unclear [8–14]. Currently, treatment options include noninvasive therapy such as medications, multiple physical modalities, and multidisciplinary biopsychosocial rehabilitation; interventional treatments, such as intradiscal radiofrequency and epidural injections; regeneration by injecting solutions of papain and methylene blue into the disc; and surgical approaches, such as intervertebral fusion or artificial disc replacement. Despite advances in pain relief therapies, they provide only temporary relief and are associated with complications [15].

IDD progresses due to cellular and biochemical changes in the IVD microenvironment, resulting in progressive functional and structural damage. The main pathological features of IDD include the production of proinflammatory mediators, progressive loss of ECM, increased cellular senescence and apoptosis, and phenotypic changes in healthy NP cells [13, 14, 16, 17]. Many molecular biology studies have demonstrated increased expression of inflammatory mediators such as IL-1*β*, TNF-*α*, IL-6, IL-8, and IL-20 in degenerative IVD [18–23]. Increased plasma inflammatory mediator concentrations are related to the degree of IDD and the severity of LBP [24]. Advances in inflammatory mediator mechanisms will significantly promote the translation of molecular research into clinical practice, offering new paths for developing IDD medication. This review is aimed at discussing the research on the potential function of inflammatory mediators in IDD.

## 2. Upstream and Downstream Regulatory Networks

**Figure 1:**
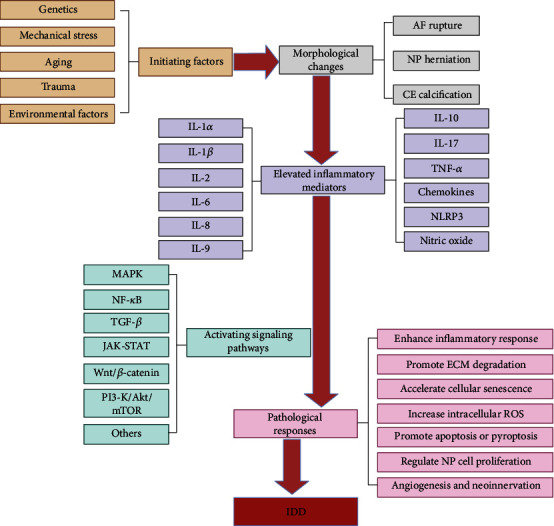
Diagram of IDD upstream and downstream regulation networks.

Disc degeneration was derived from several initializing factors, such as genetics, mechanical stress, aging, trauma, and environmental factors [25–29]. These initializing factors lead to morphological changes in the disc tissue and surrounding structures, including a series of changes such as rupture of the annulus fibrosus (AF), NP herniation, and calcification of the cartilage endplates (CE). Since the intervertebral disc is a nearly wholly enclosed avascular tissue with few sources of nutrition, accumulation of degraded organelles and waste materials that are difficult to metabolize occurs, and a closed acidic environment gradually develops, leading to an imbalance in the internal and external environment, which propagates inflammatory signals and causes a massive release of inflammatory mediators [1], including IL-1*α*, IL-1*β*, IL-2, IL-6, IL-8, IL-9, IL-10, IL-17, TNF-*α*, chemokines, the NLRP3 inflammasome, and nitric oxide. These inflammatory mediators can activate signalling pathways, such as the NF-*κ*B, PI3-K/Akt/mTOR, TGF-*β*, JAK-STAT, Wnt/*β*-catenin, and MAPK pathways, resulting in a range of pathological responses within the IVD, including an enhanced inflammatory response, promote ECM degradation, accelerate cellular senescence, increased intracellular ROS, promotion of apoptosis or pyroptosis, regulation of NP cell proliferation, and increased angiogenesis and neoinnervation. Ultimately, this process exacerbates the development of IDD. A schematic diagram of this pathological process is shown in Figure 1.

## 3. Sources of Inflammatory Mediators

**Figure 2:**
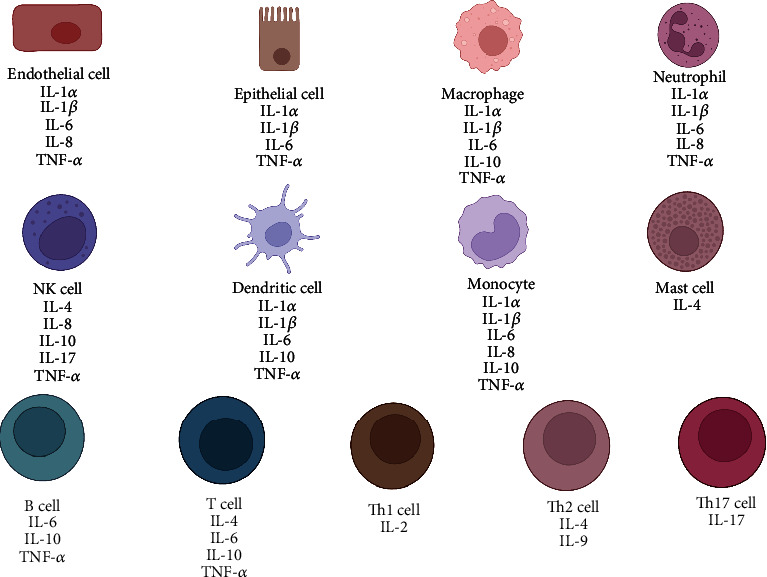
Schematic illustration of different cells expressing various cytokines.

Inflammatory mediators can be secreted by endogenous intervertebral disc cells and exogenous immune cells [30]. The normal aging process associated with genetic susceptibility leads to degeneration of the IVD, causing alterations in the ECM, such as a reduced number of functional cells, reduced proteoglycan content, malnutrition, dehydration, matrix breakdown, and calcification. Modifications in the ECM affect the typical response of the IVD to mechanical loading. The IVD becomes prone to microfissures and consequent ingrowth of nerve tissue and blood vessels. Fragments and microcrystals of the ECM may internally cause an inflammatory response, stimulating endogenous IVD cells to produce proinflammatory mediators such as IL-1*β*, IL-6, and IL-8, further promoting a chain reaction of tissue degeneration. In addition, NP is recognized by the immune system as nonself when exposed to tissues, such as through microfissures or protrusions, thereby recruiting inflammatory cells such as macrophages, endothelial cells, B cells, and T cells. These inflammatory cells can secrete inflammatory mediators. A brief overview of the various cells expressing different cytokines is presented in Figure 2.

## 4. Inflammatory Mediators

Table 1 shows the inflammatory mediators associated with IDD.

### 4.1. Interleukin (IL)

#### 4.1.1. IL-1*α*

**Figure 3:**
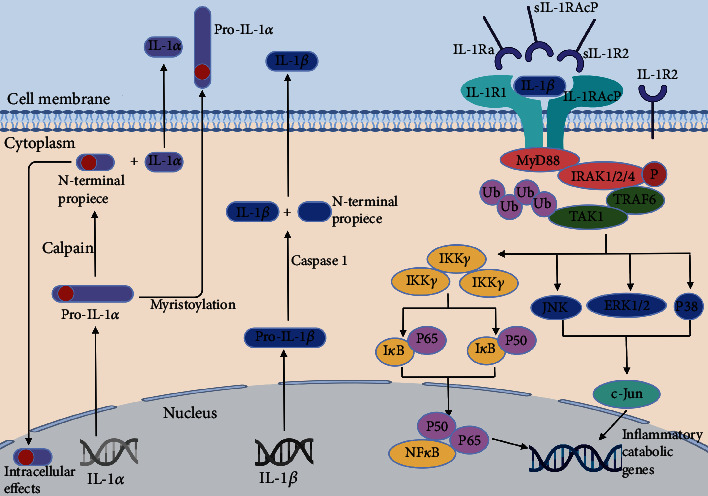
IL-1*α* and IL-1*β* synthesis and signal transduction pathways.

IL-1*α* is a critical inflammatory mediator primarily released by monocytes, macrophages, dendritic cells, and endothelial cells [31]. IL-1*α* and IL-1*β* act in the same way, and their receptors share the same ligand binding and signal transduction pathways [32]. However, unlike IL-1*β*, IL-1*α* activity is not dependent on the inflammasome caspase-1 pathway [33]. Several studies have found that IL-1*α* levels in degenerative lumbar disc tissue are elevated compared with those in normal lumbar disc tissue and that IL-1*α* levels are positively associated with the severity of IDD [31, 34]. Previous meta-analyses revealed that the IL-1*α* (+889C/T) polymorphism was related to the increased incidence of IDD in Caucasian and Chinese Han populations [35, 36]. IL-1*α* has been found to accelerate IDD development by increasing extracellular matrix-degrading enzyme production and inhibiting extracellular matrix synthesis [37, 38]. IL-1*α* may also play a role in cartilage endplate degeneration by regulating MMP-3 and TIMP-3[39]. Furthermore, IL-1*α* could contribute to LBP by inducing IVDs to produce prostaglandin E2 and other inflammatory chemicals [40]. The sensitivity of bradykinin can be enhanced by IL-1*α*, which directly irritates nerve roots and hence contributes to IDD-induced neuralgia [41]. The synthesis and signal transduction pathways of IL-1*α* and IL-1*β* are shown in Figure 3. In conclusion, IL-1*α* is of paramount importance in the development of IDD.

Two distinct genes encode IL-1*α* and IL-1*β*. Both proteins are produced as propeptide precursors (pro-IL-1*α* and pro-IL-1*β*). Pro-IL-1*α* is a physiologically active molecule with intracellular and extracellular effects. Pro-IL-1*α* has a nuclear localization sequence at its N-terminus and exists in high quantities in the nucleus. Pro-IL-1*α* is also produced as a membrane-bound cytokine after myristoylation, where it is most likely engaged in cell–cell interactions. Less frequently, the precursor form can be cleaved by a calpain-like protease to generate secreted IL-1*α* and an N-terminal peptide. Pro-IL-1*α* and the N-terminal peptide can be physiologically active after nuclear translocation. Caspase 1 cleaves pro-IL-1*β* into IL-1*β*, which may be released as a soluble, functional protein. Pro-IL1*α*, IL-1*α*, and IL-1*β* can all bind to IL1R1, allowing the recruitment of the IL1RAcP coreceptor. A series of events downstream of the IL-1R complex activate essential signalling proteins, such as mitogen-activated kinases (JNK, p38, and ERK1/2) and transcription factors, such as NF-*κ*B (p65 and p50 subunits) and c-Jun (an AP-1 subunit), which regulate the expression of several inflammatory and catabolic genes. Signalling through the IL-1R complex can be modulated by the inhibitory effects of IL-1R2, sIL-1R2, sIL-1RAcP, and IL-1Ra.

#### 4.1.2. IL-1*β*

IL-1*β* is a crucial inflammatory mediator with a wide range of actions and activities on various cells that can lead to various inflammatory processes. Systemically, IL-1*β* signalling generates an acute phase response, hypotension, vasodilation, and fever; locally, IL-1*β* signalling leads to an increase in adhesion molecules, which increases lymphocyte recruitment and amplifies the inflammatory response [42]. IL-1*β* expression has been demonstrated to be significantly increased in degenerative IVDs and is related to symptoms of LBP [43–46].

**Figure 4:**
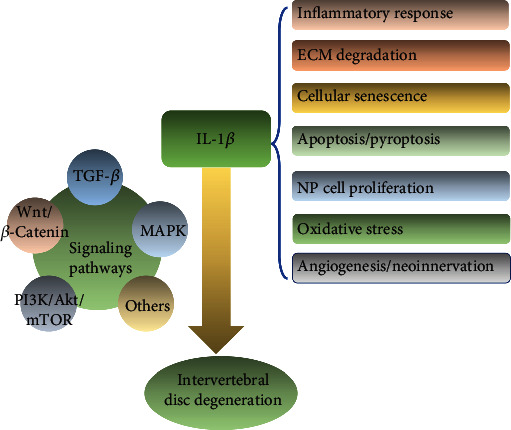
IL-1*β* is involved in multiple pathological processes of intervertebral disc degeneration.

As shown in Figure 4, IL-1*β* may influence the development of IDD through several mechanisms. First, IL-1*β* can enhance the inflammatory response of the IVD by increasing the production of inflammatory mediators, such as IL-6, IL-8, IL-17, prostaglandin E2, chemokines, and the NLRP3 inflammasome [47–50]. Second, IL-1*β* regulates ADAMTS and MMP production in the IVD, resulting in ECM degradation [38, 51–53]. Third, the output of senescence-associated-galactosidase (SA-*β*-Gal) can be enhanced by IL-1*β*, indicating that this inflammatory mediator may accelerate IDD development by hastening cellular senescence [54–57]. Fourth, IL-1*β* can promote apoptosis and pyroptosis in NP cells by regulating the NF-*κ*B and MAPK pathways, which hastens the development of IDD [50, 53, 58, 59]. Fifth, it was demonstrated that IL-1*β* regulated NP cell proliferation leading to the development of IDD [56, 60]. Additionally, IL-1*β* increases intracellular reactive oxygen species (ROS), and excessive ROS accumulation can lead to oxidative stress and the progression of IDD [61–63]. Finally, IL-1*β* might increase angiogenesis and neoinnervation inside IVDs by increasing the synthesis of vascular endothelial growth factor (VEGF), nerve growth factor (NGF), and BDNF [64, 65]. In conclusion, IL-1*β* plays a significant role in IDD and may be a promising therapeutic target.

#### 4.1.3. IL-2

IL-2, found on 4q27, is mainly generated by mature T cells and acts as a growth factor for T and B cells, playing a role in their growth. IL-2 is increased in individuals with lumbar disc herniation and influences human NPC proliferation, apoptosis, and ECM degradation through the MAPK pathway [66]. Furthermore, IL-2 gene variations have been revealed as susceptibility factors for IDD, indicating that IL-2 may play a role in the development of IDD [67]. In conclusion, IL-2 has a function in IDD, but the exact mechanism is still unclear.

#### 4.1.4. IL-4

IL-4 is a cytokine produced by T cells that regulates the activity of various immune cells. IL-4 is primarily generated by immune cells, but its receptors are found in various cell types and promote cell proliferation and differentiation, tissue regeneration, and neurological function. It was discovered that IL-4 expression was significantly higher in IDD patients than in healthy controls [68–70]. Interestingly, unlike IL-1, IL-4 exhibits direct anti-inflammatory actions by binding to the IL-4RA receptor on 16p12.1 and blocking the induction pathway of IL-1 and TNF-*α* [71–75]. In conclusion, IL-4 performs an anti-inflammatory function in IDD and can be used to treat this disorder.

#### 4.1.5. IL-6

IL-6 is an important cytokine that can be secreted by T cells, macrophages, and NP cells. According to research, patients with disc degeneration have higher serum IL-6 levels than healthy controls [76, 77]. It has also been demonstrated that increased serum IL-6 levels are associated with disc degeneration-related LBP [78, 79]. Furthermore, IL-6 levels are linked to the degree of disc degeneration and pain intensity [80–83]. There are multiple potential mechanisms for IL-6 involvement in IDD. IL-6 accelerates the course of IDD by increasing the catabolic effects of IL-1*β* and TNF-*α* on NP cells through the JAK/STAT signalling pathway [84]. Moreover, IL-6 promotes apoptosis of neurons in the dorsal root ganglion, resulting in sensory impairment [85]. Furthermore, IL-6 promotes the degeneration of NP cells by blocking miR-10a-5p and hence the IL-6R signalling pathway, which in turn encourages chondrocyte ferrogenesis [86]. In conclusion, IL-6 plays an essential role in IDD and may be a target for future therapy.

#### 4.1.6. IL-8

IL-8 is a chemokine with a distinct CXC amino acid sequence [87]. IL-8 expression is considerably higher in the disc tissue of IDD patients, indicating that it may have a role in the disease [88–90]. IL-8 can activate microglia in the spinal cord, promote the upregulation of neuroinflammatory markers such as IL-1*β* and TNF-*α*, and exacerbate the inflammatory response, aggravating the development of IDD [91]. IL-8 can also regulate angiogenesis by enhancing extracellular matrix survival, proliferation, and MMP-2 production through the MAPK signalling pathway, thereby affecting IDD progression [87, 92, 93].

#### 4.1.7. IL-9

IL-9 is a polymorphic cytokine that regulates the Th2 inflammatory response [94]. IL-9 was shown to upregulate TNF-*α* and PGE2 production in NP cells, and its blood levels were positively associated with the degree of disc degeneration in IDD patients [95]. Therefore, IL-9 may play a role in the autoimmune inflammatory process in IDD, but the exact mechanism is not yet clear.

#### 4.1.8. IL-10

Interleukin-10 (IL-10) is an important immune system regulator that regulates inflammation and tissue hemostasis [96]. IL-10 SNPs have been linked to IDD, suggesting that genetic alterations in IL-10 may lead to intervertebral disc imbalances and degeneration [97]. The expression of IL-10 is considerably higher in IDD patients, indicating the close relationship between this inflammatory cytokine and the disorder [70, 77]. Furthermore, in IDD animal models, IL-10 expression levels in several spinal components (bone, discs, and ligaments) were dramatically upregulated [98]. According to previous studies, IL-10 may hasten IDD development by intensifying the inflammatory response [99, 100]. To summarize, IL-10 plays a role in the degenerative process of IDD and can potentially be a new therapeutic target.

#### 4.1.9. IL-17A

IL-17 is a cytokine primarily generated by the T helper 17 subsets of CD4+ T cells and plays a vital role in various inflammatory disorders [101, 102]. It has six members in its family, from IL-17A to IL-17F [103]. IL-17A, one of the most important members of the IL family, has been related to a range of degenerative illnesses [104, 105]. It has been demonstrated that IL-17A is more abundant in degenerative disc tissue than in normal tissue [96, 106, 107]. There are various probable theories for the mechanism of action. In NP cells, IL-17A can increase the production of inflammatory markers, such as IL-6, COX-2, MMPs, IFN-*γ*, and TNF-*α* [106, 108–110]. IL-17A has been found to regulate the development of IDD by modulating the ECM metabolism balance linked with ADAMTS-7 expression [107, 111, 112]. In addition, IL-17A may accelerate the development of IDD by blocking autophagy in human degenerative NP cells through stimulation of the PI3K/Akt/Bcl-2 signalling pathway [113, 114]. To summarize, the involvement of IL-17 in IDD is significant, and it may be an essential target for IDD treatment.

### 4.2. TNF-*α*

Tumour necrosis factor-alpha (TNF-*α*), located at 6p21.33, is mainly synthesized as a transmembrane protein and is turned into an active molecule following processing by specific enzymes, including TNF-*α* converting enzymes [115]. TNF-*α* is a proinflammatory cytokine linked to some pathological illnesses, including infections, autoimmune diseases, cancer, atherosclerosis, Alzheimer's disease, and inflammatory bowel disease [116–121]. TNF-*α* also regulates various developmental and immunological processes, including inflammation, differentiation, lipid metabolism, and apoptosis [122–124]. TNF-*α* has been linked to almost every component of the human immune system [125].

Studies have shown that TNF-*α* expression is upregulated in degenerative disc tissue more than in normal tissue [126–129]. TNF-*α* levels were also found to be positively associated with the severity of IDD [129–131]. In the absence of substantial deterioration, transgenic mice overexpressing human TNF-*α* exhibited early onset spontaneous disc herniation [132]. In a porcine model, lumbar discs treated with exogenous TNF-*α* displayed degenerative alterations, including annular fissures, loss of NP matrix, vascularization, and expression of IL-1*β* in the outer annulus, indicating that TNF-*α* is a driver of disc degeneration [133].

**Figure 5:**
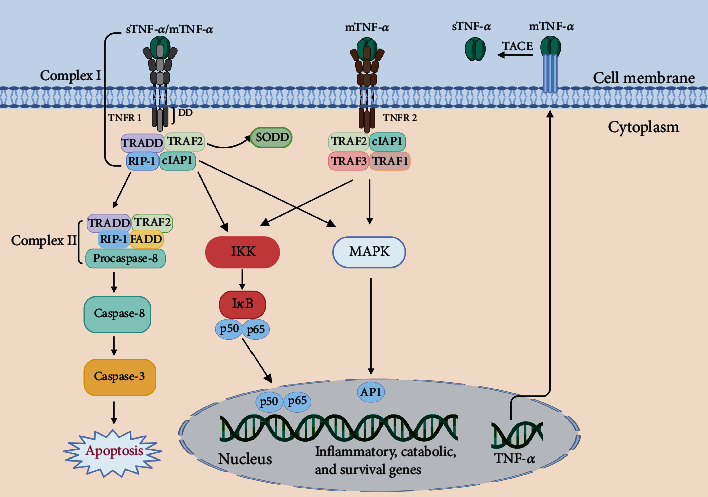
TNF-*α* signalling pathway.

As shown in Figure 5, TNF-*α* binds to two receptors: TNF receptor type 1 (TNFR1) and TNF receptor type 2 (TNFR2). TNF-*α* may be implicated in IDD in many ways. TNF-*α* has been demonstrated in multiple studies to trigger IVDs by releasing many proinflammatory cytokines, including IL-1, IL-6, IL-8, IL-17, NO, and PGE2, and chemokines, which further exacerbate the inflammatory response of discs [134–137]. TNF-*α* also increases the synthesis of substance P, NGF, and VEGF, all of which can cause pain by sensitizing the nervous system and driving neurovascular development toward IVD [138, 139]. Furthermore, TNF-*α* stimulates ECM breakdown mostly via the NF-*κ*B/MAPK signalling pathway [140–144]. TNF-*α* also interacts with its receptor and affects the JNK/ERK-MAPK and NF-*κ*B signalling pathways in NPCs during IDD, upregulating proapoptotic proteins and downregulating antiapoptotic proteins, resulting in apoptosis [145–149]. Furthermore, TNF-*α* has been shown to cause premature senescence in NPCs [150, 151]. Additionally, TNF-*α* can affect the proliferation of NP cells via the JNK, NF-*κ*B, Notch, UPR/XBP1, and p38 MAPK signalling pathways [152–156].

TNF-*α* is generally found as a stable homotrimer known as mTNF-*α*. TACE, a metalloproteinase, can convert mTNF-*α* to sTNF-*α*. TNF-*α* works via two distinct receptors, TNFR1 and TNFR2. sTNF-*α* or mTNF-*α* may bind to transmembrane TNFR1, resulting in a conformational shift and release of the inhibitory SODD protein. Bound TNFR1 recruits several factors, including TRADD, RIP1, TRAF2, and cIAP 1 and 2, to form complex I, which signals via the NF-*κ*B or MAPK pathway, and activate p65 or AP1. Complex I signalling causes inflammation (through chemokines and cytokines) and activates stromal catabolic genes (MMPs and ADAMTSs), as well as survival-promoting genes (cIAP1 and 2, cFLIP, TRAF1, and TRAF2). In addition, mTNF-*α* may also activate TNFR2, resulting in a similar complex and downstream signaling cascade. In specific circumstances, TNFR1 bound to sTNF-*α* may be internalized into complex II, causing procaspase 8 to be converted into caspase 8 and then caspase 3 to be activated, eventually leading to apoptosis.

### 4.3. Chemokines

Chemokines are significant second-order cytokines produced in response to stimuli and play an essential role in acute and chronic inflammation [134]. Based on the primary cysteine residues involved in disulphide bonding, chemokines have been categorized as C, CC, CXC, and CX3C [157]. According to a bioinformatics study, numerous chemokine genes may have a role in the development of IDD caused by inflammatory reactions [158]. CCL2, CCL5, CXCL6, CXCL12, CXCL20, C-X-C receptor 4 (CXCR4), and stromal cell-derived factor 1 (SDF1) expression is considerably elevated in IDD tissues [159–163]. Serum CCL3, CXCL12, and SDF1 levels have also been demonstrated to be positively associated with the degree of IDD [137, 162, 164, 165]. Chemokines may have a role in IDD through a variety of pathways. Zhang et al. [166] discovered that the CCL20/CCR6 pathway attracts IL-17-producing cells to degenerate IVDs and that IL-17 is implicated in the autoimmune process of IDD in a rat model. Furthermore, CXCL12 promotes ECM disintegration and enhances MMP production in human disc endplate chondrocytes [167]. SDF1/CXCR4 was discovered to be higher in degenerating intervertebral discs, and it promotes apoptosis of NPCs via the NF-B pathway, leading to IDD [168]. Furthermore, the SDF1/CXCR4 axis, via the PI3K/AKT pathway, can regulate VEC survival, migration, tube formation, and angiogenesis in human degenerative discs [169–171].

### 4.4. The NLRP3 Inflammasome

The NLRP3 inflammasome is a multiprotein complex in the cytoplasm that consists of a receptor, adaptor, and effector [172]. NLRP3 expression in IDD was observed to be considerably higher than that in normal disc tissue [173, 174]. There is further evidence from MRI and histology that NLRP3 is linked to the progression of IDD [175]. It has been demonstrated that overactivation of the NLRP3 inflammasome results in the overproduction of downstream IL-1, which is vital in the development of IDD [173]. Activation of the NLRP3 inflammasome can also cause apoptosis in NP cells [176, 177]. In addition, *Propionibacterium acnes* can activate the NLRP3 inflammasome via the TXNIP-NLRP3 pathway, causing pyroptosis of NP cells and IDD [178]. In summary, the NLRP3 inflammasome plays a crucial role in IDD, and more research is needed to discover its mechanism of action.

### 4.5. Nitric Oxide

**Figure 6:**
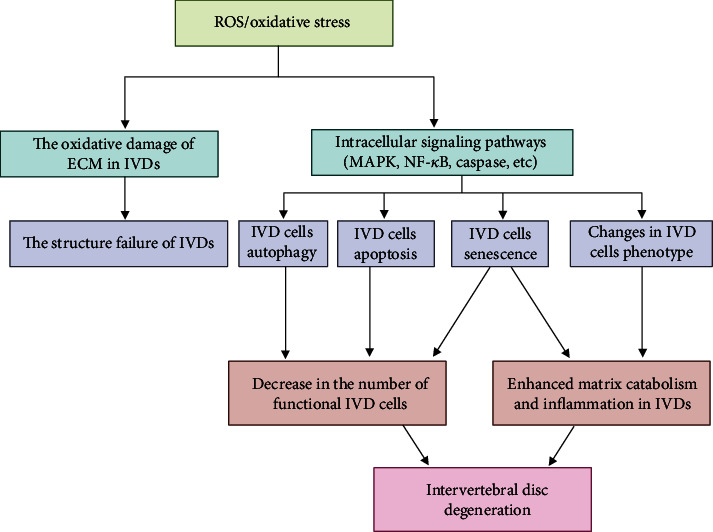
The role of ROS/oxidative stress in the development of IDD.

NP cells can create nitric oxide (NO), and it was shown that NO production is enhanced in IDD and that its synthesis relies on nitric oxide synthase (NOS) [131]. TNF-*α*, IL-1*β*, lipopolysaccharide, and interferon-*γ* were discovered to promote NO production [89, 179]. Nitric oxide has proinflammatory effects, and its role as a vasodilator promotes vascular leakage, inhibits proteoglycans, and induces neuropathic pain, all of which contribute to IDD [180]. In addition, NO is regarded as a member of the ROS superfamily due to its similar effects to those of ROS, and ROS hasten intervertebral disc degeneration. The specific mechanism is shown in Figure 6.

ROS alter the ECM of IVDs through oxidative modification, eventually impairing the structure of IVDs. ROS activate multiple signaling pathways, such as the MAPK and NF-*κ*B pathways, thereby regulating autophagy, apoptosis, senescence, and the phenotype of IVD cells, thus reinforcing matrix degradation and inflammation and enhancing the decrease in the number of functional IVD cells. Ultimately, ROS/oxidative stress promotes the progression of IDD.

## 5. Therapeutic Prospects for IDD by Targeting Inflammation

The inflammatory response that mediates the degenerative cascade in IVDs is being targeted as a potential therapeutic or prognostic strategy. Currently, the main goals of therapies are to manage degenerated IVDs and relieve symptoms. The conventional approaches include systemic medicine and surgical decompression/discectomy. However, these methods are not aimed at the pathogenesis of IDD. In this section, we focused on reviewing and providing more information on novel anti-inflammation therapies for IDD, including intradiscal injections, gene therapies, MSC-based therapies, and exosome-based therapies.

### 5.1. Intradiscal Injections

Injecting medications into the IVD is one of the most straightforward ways to regulate inflammation in IVDs. TNF-*α* inhibitors are examples of medications administered in this way [181]. TNF inhibitors, such as infliximab and Atsttrin, have been shown to decrease the inflammatory response [182, 183]. Infliximab is an antibody against TNF-*α*. Injecting infliximab into the IVD of rats alleviated discomfort compared with the control groups [184]. Atsttrin is an inflammatory-related growth factor consisting of three pieces of progranulin. In a mouse model, this protein inhibited TNF-initiated inflammatory signaling by binding directly to TNF-*α* receptors [185]. Additionally, Atsttrin suppressed TNF-induced inflammatory cytokine production, including production of MMP-13, COX-2, iNOS, and IL-17, causing concomitant catabolic alterations in cartilage, disc height, and NP cells in ex vivo cultured rat discs [183].

The IL-1 inhibitor, IL-1 receptor antagonist (IL-1Ra), binds to the IL-1 receptor (IL-1R) and blocks the transmission of inflammatory signals [141]. IL-1Ra may have a therapeutic role in IDD, according to previous studies [38, 186, 187]. Injection of IL-1Ra into both degenerative and nondegenerative human IVD tissues reduced the production of matrix breakdown proteases, such as type II collagenase, gelatinase, and caseinase [38]. Another study revealed the therapeutic efficacy of IL-1Ra by applying polylactic-co-glycolic acid (PLGA) microspheres as a delivery vehicle. In NP cell cultures, IL-1Ra-PLGA microspheres attenuated the degradative effects of IL-1*β* on NP cells by suppressing NO production while restoring the levels of iNOS, IL-6, ADAMTS-4, and MMP-13 [186].

COX-2, which controls PGE2 production in inflammatory circumstances, is also a target for suppressing inflammation in IVDs [188]. In a rat model of disc herniation, epidural injections of COX-2 inhibitors resulted in satisfactory pain relief [189]. Additionally, the inhibitor of IkB kinase-b (IKKb), which is involved in NF-kB activation, is a novel candidate for treating inflammation in IVD. Intradiscal injection of IKKb downregulated the expression of TNF-*α*, IL-1*β*, and IL-6 in degenerative discs and neuropeptides in dorsal root ganglion neurons [190]. Despite promising results, injection of such molecules in IVDs may be ineffective owing to their short half-life and the complicated microenvironment of degenerative IVDs [30]. Furthermore, the potential risk of IDD caused by puncturing should be noted.

The injection of phytochemicals derived from medicinal plants has been researched in recent years because of its cost-effectiveness and biological functions. According to previous in vivo and in vitro studies, various phytochemicals, including resveratrol [191], mangiferin [192], epigallocatechin-3-gallate [177], chlorogenic acid [193], celastrol [194], isofraxidin [195], higenamine [196], sesamin [197, 198], honokiol [176], naringin [199, 200], baicalein [201], berberine [53], wogonin [52], and luteoloside [202]. Most of these phytochemicals inhibit the IL-1*β*-induced or TNF-*α*-induced inflammatory response and extracellular matrix degradation in NP cells. Although satisfactory therapeutic effects of phytochemicals in IDD have been reported, the metabolic processes, organ distribution, and toxicity of different doses still need to be investigated.

### 5.2. Gene Therapies

With the ability of locally modifying the expression of a certain gene and production of the corresponding protein, gene therapy offers longer sustained effects in IDD [203]. A study published in 1997 proposed genetic modifications as a positional treatment for IDD [204]. In this study, a retrovirus vector was developed to transduce IL-1Ra into bovine chondrocyte cells. Injection of cells overexpressing IL-1Ra significantly downregulated MMP3 for 14 days in degenerative IVD tissue, reducing IL-1-mediated matrix degradation and halting the deterioration of IDD. In a rabbit model, NP cells transfected with TGF-*β*1 demonstrated increased proteoglycan production [205]. Consistent with this finding, TGF-*β*1-transfected senescent NP cells of humans also enhanced the synthesis of proteoglycan and collagen [206, 207].

The safety of gene therapy may restrict its application in clinical settings. For the treatment of chronic IDD, high dosage exposure and long-term usage may induce oncogenesis, which is a critical concern [208]. Improvement in the reliability of viral vector designs and expression control of transgenes might allow the safe use of gene therapy.

### 5.3. MSC-Based Therapies

In recent years, many cell-based treatments to regenerate IVDs have been developed [209, 210]. Among the candidates, MSCs have the best potential for IVD regeneration, which is attributed to their autologous transplantation ability [211]. MSCs boosted collagen type II expression and slowed the apoptosis process of NP cells [212]. Additionally, IVD tissue survived for 6 months in rabbits with the concomitance of MSCs [213]. However, the number of transplanted MSCs is important [214]. In addition to their multidifferentiation capability, the immunomodulatory role of MSCs has been revealed [215, 216]. MSCs participate in inflammation by releasing cytokines, which directly interact with degenerative NP cells [217]. In vitro studies showed that MSCs cocultured with rat NP cells inhibited the expression of proinflammatory cytokines, including IL-3, IL-6, IL-11, IL-15, and TNF-*α* [218]. In a clinical trial, LBP was significantly alleviated by three months of MSC injection, and the authors concluded that MSCs stimulated the regeneration of IVD and had immunomodulatory characteristics [219]. In another 2-year follow-up study, after the injection of umbilical cord-derived MSCs into IVDs, LBP and lumbar function were improved and maintained during the duration of follow-up [220]. Although benefits and promising outcomes of MSC-based therapies have been observed, the mechanisms have still not been clearly elucidated by animal experiments, and most of the clinical studies were case reports with limited sample sizes.

### 5.4. Exosome-Based Therapies

Exosomes and exosomal miRNAs have been the focus of IDD therapy in recent years. The potential mechanisms reported in previous studies could be categorized as angiogenesis of the ECM, senescence, metabolic homeostasis, proliferation, apoptosis, and oxidative stress [221]. Additionally, exosomes and exosomal miRNAs also play an important role in the regulation of inflammation in IVDs [222]. By downregulating LRG1, BMSC-derived exosomal miR-129-5p attenuated the activation of the p38 MAPK pathway to inhibit macrophage polarization from the M1 to M2 phenotype, which resulted in the release of anti-inflammatory mediators and prevented apoptosis of NP cells as well as degradation of ECM [223]. NLRP3, a member of the inflammasome, is a crucial component of innate immunity and participates in several proinflammatory processes [224]. NLRP3 can be extremely upregulated in the development of IDD [225]. By blocking the NLRP3/caspase-1 pathway, MSC-derived exosomal miR-410 reversed the expression of IL-1*β* and IL-18, reducing LPS-induced pyroptosis in NP cells [226]. Similarly, human umbilical cord mesenchymal stem cell- (hucMSC-) derived miR-26a-5p affected mRNA methyltransferase (METTL14) and m6A methylation in NP cells, which downregulated the expression of NLRP3, leading to the inhibition of pyroptosis and the release of proinflammatory cytokines [227]. As a novel therapy, more studies focused on the role of exosomes in IDD treatment are expected.

## 6. Conclusion

IDD is a prevalent musculoskeletal illness that produces LBP and negatively impacts quality of life. Recent research has revealed that various inflammatory mediators, such as IL-1*β*, TNF-*α*, IL-6, IL-17, chemokines, and the NLRP3 inflammasome, play an essential role in IDD. Most research has found that inflammatory mediators have a role in the development of IDD primarily through the control of the inflammatory response, IVD cell proliferation, senescence, apoptosis, pyroptosis, autophagy, ECM degradation, and oxidative stress. Targeting these inflammatory mediators may lead to future optimum IDD treatment. Clinical investigations have recently revealed that inhibiting IL-1*β* and TNF-*α* is a promising future therapy for IDD. More research into IDD-related inflammatory mediators is needed to help us understand the molecular pathophysiology of IDD and provide novel ideas for future IDD therapy based on inflammatory mediators.

## Figures and Tables

**Table 1:** Inflammatory mediators associated with IDD progression.

Name	Size (amino acids)	Chromosomal location	Gene	Origins	Receptor	Function in IDD	Signaling pathways
IL-1*α*	271	2q14	IL1A (IL1F1)	Neutrophil; macrophage; monocyte; endothelial cell; epithelial cell; dendritic cell	IL1R1; IL1R2	Inhibit ECM synthesis; enhance inflammatory response; enhance bradykinin sensitivity	TGF-*β*; MAPK; NF-*κ*B; Wnt/*β*-catenin; PI3K/Akt/mTOR
IL-1*β*	269	2q14	IL1B (IL1F2)	Neutrophil; macrophage; monocyte; endothelial cell; epithelial cell; dendritic cell	IL1R1; IL1R2	Enhance inflammatory response; promote ECM degradation; accelerate cellular senescence; promote apoptosis and pyroptosis; regulate NP cell proliferation; increase intracellular ROS; increase angiogenesis and neoinnervation	TGF-*β*; MAPK; NF-*κ*B; Wnt/*β*-catenin; PI3K/Akt/mTOR
IL-2	153	4q26-q27	IL2	Th1 cell	IL2RA; IL2RB; IL2RG	Regulate NP cell proliferation; promote apoptosis; promote ECM degradation	PI3K/Akt/mTOR; MAPK; JAK-STAT
IL-4	153	5q31.1	IL4	Th2 cell; NK cell; T cell; mast cell	IL4R	Inhibit inflammatory processes	JAK-STAT
IL-6	212	7p21	IL6 (IFNB2)	Neutrophil; macrophage; T cell; B cell; monocyte; endothelial cell; epithelial cell; dendritic cell	IL6R	Enhance inflammatory response; promote apoptosis of neurons in the dorsal root ganglion; promote chondrocyte ferrogenesis	JAK-STAT; MAPK; PI3K/Akt/mTOR
IL-8	99	4q13-q21	CXCL8 (IL8)	Neutrophil; NK cell; endothelial cell; monocyte	CXCR1; CXCR2	Enhance inflammatory response; regulate angiogenesis	MAPK; JAK-STAT; NF-*κ*B; PI3K/Akt/mTOR
IL-9	144	5q31.1	IL9	Th2 cell	IL9R	Enhance inflammatory response	MAPK; JAK-STAT
IL-10	178	1q31-q32	IL10	Macrophage; T cell; B cell; NK cell; monocyte; dendritic cell	IL10R1; IL10R2	Enhance inflammatory response	MAPK; JAK-STAT; NF-*κ*B
IL-17A	155	6p12	IL17A (CTLA8, IL17)	Th17 cell; NK cell	IL17RA; IL17RC	Enhance inflammatory response; promote ECM degradation; block autophagy in degenerating NP cells	MAPK; NF-*κ*B; C/EBP*β*/*δ*
TNF-*α*	233	6p21.33	TNF (TNFA, TNFSF2)	Endothelial cell; epithelial cell; macrophage; neutrophil; NK cell; dendritic; monocyte; B cell; T cell	TNFR1; TNFR2	Enhance inflammatory response;1 promote ECM degradation;1 accelerate cellular senescence;1 promote apoptosis;1 regulate NP cell proliferation;1 increase angiogenesis and neoinnervation	MAPK; NF-*κ*B; Notch; UPR/XBP1
Chemokines	-	-	-	Endothelial cell; epithelial cell; B cell; T cell; NK cell	CR; CCR; CXCR; CX3CR	Enhance inflammatory response; promote ECM degradation; promote apoptosis; Increase angiogenesis and neoinnervation	NF-*κ*B; PI3K/Akt/mTOR
NLRP3	1036	1q44	NLRP3 (C1orf7, CIAS1, NALP3, PYPAF1)	Neutrophil; macrophage	-	Enhance inflammatory response; promote apoptosis	NF-*κ*B

## Data Availability

The data used to support the findings of this study are available from the corresponding authors upon request.
